# Hyperglycemia Aggravates Hepatic Ischemia and Reperfusion Injury by Inhibiting Liver-Resident Macrophage M2 Polarization *via* C/EBP Homologous Protein-Mediated Endoplasmic Reticulum Stress

**DOI:** 10.3389/fimmu.2017.01299

**Published:** 2017-10-13

**Authors:** Zhuqing Rao, Jie Sun, Xiongxiong Pan, Ziyang Chen, Heliang Sun, Panpan Zhang, Mei Gao, Zhengnian Ding, Cunming Liu

**Affiliations:** ^1^Department of Anesthesiology, First Affiliated Hospital with Nanjing Medical University, Nanjing, China

**Keywords:** liver ischemia and reperfusion, hyperglycemia, macrophage, Kupffer cell, endoplasmic reticulum stress, C/EBP homologous protein

## Abstract

Aggravated liver ischemia and reperfusion (IR) injury has been observed in hyperglycemic hosts, but its underlying mechanism remains undefined. Liver-resident macrophages (Kupffer cells, KCs) and endoplasmic reticulum (ER) stress play crucial roles in the pathogenesis of liver IR injury. In this study, we evaluated the role of ER stress in regulating KC activation and liver IR injury in a streptozotocin-induced hyperglycemic/diabetic mouse model. Compared to the control group (CON group), hyperglycemic mice exhibited a significant increase in liver injury and intrahepatic inflammation following IR. KCs obtained from hyperglycemic mice secreted higher levels of the pro-inflammatory factors TNF-α and IL-6, while they secreted significantly lower levels of the anti-inflammatory factor IL-10. Furthermore, enhanced ER stress was revealed by increased C/EBP homologous protein (CHOP) activation in both IR-stressed livers and KCs from hyperglycemic mice. Specific CHOP knockdown in KCs by siRNA resulted in a slight decrease in TNF-α and IL-6 secretion but dramatically enhanced anti-inflammatory IL-10 secretion in the hyperglycemic group, while no significant changes in cytokine production were observed in the CON group. We also analyzed the role of hyperglycemia in macrophage M1/M2 polarization. Interestingly, we found that hyperglycemia inhibited IL-10-secreting M2-like macrophage polarization, as revealed by decreased *Arg1* and *Mrc1* gene induction accompanied by a decrease in STAT3 and STAT6 signaling pathway activation. CHOP knockdown restored *Arg1* and *Mrc1* gene induction, STAT3 and STAT6 activation, and most importantly, IL-10 secretion in hyperglycemic KCs. Finally, *in vivo* CHOP knockdown in KCs enhanced intrahepatic anti-inflammatory IL-10 gene induction and protected the liver against IR injury in hyperglycemic mice but had no significant effects in control mice. Our results demonstrate that hyperglycemia induces hyper-inflammatory activation of KCs during liver IR injury. Thus, hyperglycemia-induced CHOP over-activation inhibits IL-10-secreting M2-like macrophage polarization by liver-resident macrophages, thereby leading to excessive inflammation and the exacerbation of liver IR injury in diabetic/hyperglycemic hosts. This study provides novel mechanistic insight into macrophage inflammatory activation under hyperglycemic conditions during liver IR.

## Introduction

Liver ischemia and reperfusion (IR) injury is a frequent complication in patients subjected to major hepatic surgeries such as partial hepatectomy and liver transplantation (1). Liver inflammation caused by the innate immune response of macrophages plays a critical role in the pathogenesis of liver IR injury. Innate immune cells, such as Kupffer cells (KCs), dendritic cells, and natural killer cells, initiate local inflammation and recruit circulating monocytes and polymorphonuclear cells into IR-damaged livers, further amplifying local tissue injury (2).

Diabetes is a global epidemic, and up to 25% of liver transplant patients have pre-existing diabetes mellitus (3). Pretransplant diabetes is a predictor of poor outcome following liver transplantation (4). One recent study reported that patients with diabetes have a higher risk of liver graft rejection (5). Diabetes mellitus is also a major risk factor involved in ischemic diseases affecting multiple organs, including the heart (6), brain (7), kidney (8), and liver (9, 10). The inflammatory immune response is an important cause of transplantation rejection. Hyperglycemia, the most prominent sign of diabetes, has been shown to trigger chronic inflammation (11). Hallmarks of diabetes such as hyperlipidemia and hyperglycemia could induce epigenetic changes that promote an inflammatory macrophage phenotype (12). Furthermore, in hyperglycemic mice, an increased macrophage population was found in livers, kidneys, intestines, and the peritoneal cavity under inflammatory conditions (13).

Metabolic disturbance during liver IR due to hypoxia and nutrient deficiency triggers intracellular stress responses, such as the endoplasmic reticulum (ER) stress response. The unfolded protein response (UPR) is activated upon ER stress through the action of three transmembrane receptors: protein kinase R-like endoplasmic reticulum kinase (PERK), activating transcription factor 6 (ATF6), and inositol requiring kinase 1 (IRE1) (14). Critical roles of ER stress have been found to regulate liver IR injury (15, 16). Nutrition and oxygen depletion after ischemia triggered ER stress in the liver in both parenchymal and non-parenchymal cells. In addition to its roles in regulating protein-folding stress, metabolism, and cell differentiation, ER stress was recently shown to regulate innate immunity (17, 18). All three branches of the UPR directly engage inflammatory pathways through the activation of NF-κB or JNK signaling (19). The transcription factor C/EBP homologous protein (CHOP) is a downstream component of ER stress pathways. Recent studies have demonstrated the role of CHOP in regulating macrophage survival (20), pro-inflammatory activation, and polarization (21).

In diabetic hosts, the liver is often more susceptible to IR injury due to diabetes-associated microcirculation diseases and hyper-inflammation. Although the aggravation of liver IR injuries by hyperglycemia has been reported (9, 10, 22), the mechanism underlying this phenomenon is not fully defined.

In this study, we determined whether and how diabetes regulates liver IR injury and inflammatory immune activation in a streptozotocin (STZ)-induced diabetic mouse model, with a focus on the role of ER stress signaling pathways in regulating KC activation and polarization.

## Animals and Methods

### Mice

Male wild-type C57BL/6 mice (6–8 weeks old) were purchased from the Laboratory of Animal Resources of Nanjing Medical University. Animals were housed under specific pathogen-free conditions with free access with tap water and food. All animals received humane care and all animal procedures met the relevant legal and ethical requirements according to a protocol (number NMU08-092) approved by the Institutional Animal Care and Use Committee of Nanjing Medical University.

### Mouse Diabetes Model

Diabetes was induced by intraperitoneal injection of 40 mg/kg STZ dissolved in citrate buffer solution into 6-week-old mice for five consecutive days. Blood glucose levels were tested at day 14 (9 days following the last STZ injection). Mice with blood glucose over 300 mg/dL were considered hyperglycemic (STZ group). The vehicle control group (CON group) was subject to the same intraperitoneal injection procedure but with sodium citrate buffer.

### Model of Warm Liver IRI

A model of partial hepatic warm IRI was used. In brief, after successful anesthesia with 10% chloral hydrate (0.3 g/kg i.p.), mice were injected with heparin (100 mg/kg). An atraumatic clip was used to interrupt the arterial and portal venous blood supply to the cephalad lobes of the liver. The clip was removed to initiate liver reperfusion after 90 min of ischemia. All mice were placed in a designed warm container (HTP-1500 Heat Therapy Pump, Adroit Medical Systems, USA) to maintain their temperature at 29°C. Mice were sacrificed 6 h after reperfusion. Sham controls underwent the same procedure, but without vascular occlusion. Carprofen (6 mg/kg) was administered intraperitoneally for analgesia in all groups before surgery.

### *In Vivo* CHOP Knockdown

C/EBP homologous protein siRNA (Santa Cruz, CA, USA) was premixed with mannose-conjugated polymers (Polyplus transfection, USA) at a ratio specified by the manufacturer and was administered by tail vein injection (siRNA 2 mg/kg) 4 h prior to the onset of liver ischemia.

### Serum Biochemical Measurements and Liver Histopathology

Mice were sacrificed at 6 h post-reperfusion. Blood and liver samples were collected. Serum alanine aminotransferase levels were measured with an AU5400 automated chemical analyzer (Olympus, Tokyo, Japan). Liver specimens were fixed in 10% buffered formalin and embedded in paraffin. Liver sections (4 µM) were stained with H&E. The severity of liver IRI was graded blindly using Suzuki’s criteria on a scale from 0 to 4.

### TUNEL Staining

TUNEL staining of liver tissues was performed using a fluorescent detection kit (Roche Diagnostics) according to the manufacturer’s instructions.

### KC Isolation and Cell Culture

Mouse livers were perfused *in situ via* the portal vein with HBSS, followed by 0.27% collagenase IV (Sigma, Saint Louis, MO, USA). Perfused livers were dissected and teased through 70-µm cell strainers, followed by suspension in 40 mL of DMEM supplemented with 10% FBS. Non-parenchymal cells were separated from hepatocytes by centrifugation at 50 × *g* for 2 min three times. NPCs were plated in cell culture dishes in DMEM supplemented with 10% FBS, 10 mM HEPES, 2 mM GlutaMax, 100 U/mL penicillin, and 100 mg/mL streptomycin for 15 min at 37°C, then the non-adherent cells were removed. The adherent cells (KCs, 80–90% F4/80 positive) were used for further *ex vivo* experiments. KCs were cultured *in vitro* for 6 h and then cells or supernatants were collected for further analysis.

### ELISA

TNF-a, IL-6, and IL-10 levels in cell culture supernatants or serum were measured using an ELISA kit (eBiosciences, San Diego, CA, USA) according to the manufacturer’s protocols.

### Western Blots

Liver tissue or cell lysate proteins were extracted and subjected to 12% SDS-PAGE electrophoresis and transferred to a PVDF nitrocellulose membrane. Primary antibodies against cleaved-ATF6 (c-ATF6, Novus, Littleton, CO, USA), ATF4 (Proteintech Group, Chicago, IL, USA), CHOP (Cell Signaling Technology, MA, USA), spliced XBP1 (s-XBP1, Abcam, Cambridge, MA, USA), and β-actin (Cell Signaling Technology, MA, USA) were used and incubated overnight at 4°C. After 2 h of incubation with the appropriate HRP-conjugated secondary antibody (1:1,000), Clarity™ Western ECL Substrate (Bio-Rad, CA, USA) was used for chemoluminescence development. ImageJ 1.47v software was used to quantify the Western blot bands.

### Quantitative RT-PCR

Total RNA (2 µg) was reverse-transcribed to cDNA using a SuperScript III System (Invitrogen, Carlsbad, CA, USA). Quantitative PCR was performed using SYBR Green Master Mix (Roche, Indianapolis, IN, USA).

### Statistical Analysis

Results are shown as the mean ± SD. Multiple group comparisons were performed using one-way analysis of variance followed by Bonferroni’s *post hoc* test. All analyses were performed using Stata software (version 11.0). *P* values less than 0.05 (two-tailed) were considered statistically significant.

## Results

### Hyperglycemia Aggravated Liver IR Injury

**Figure 1:**
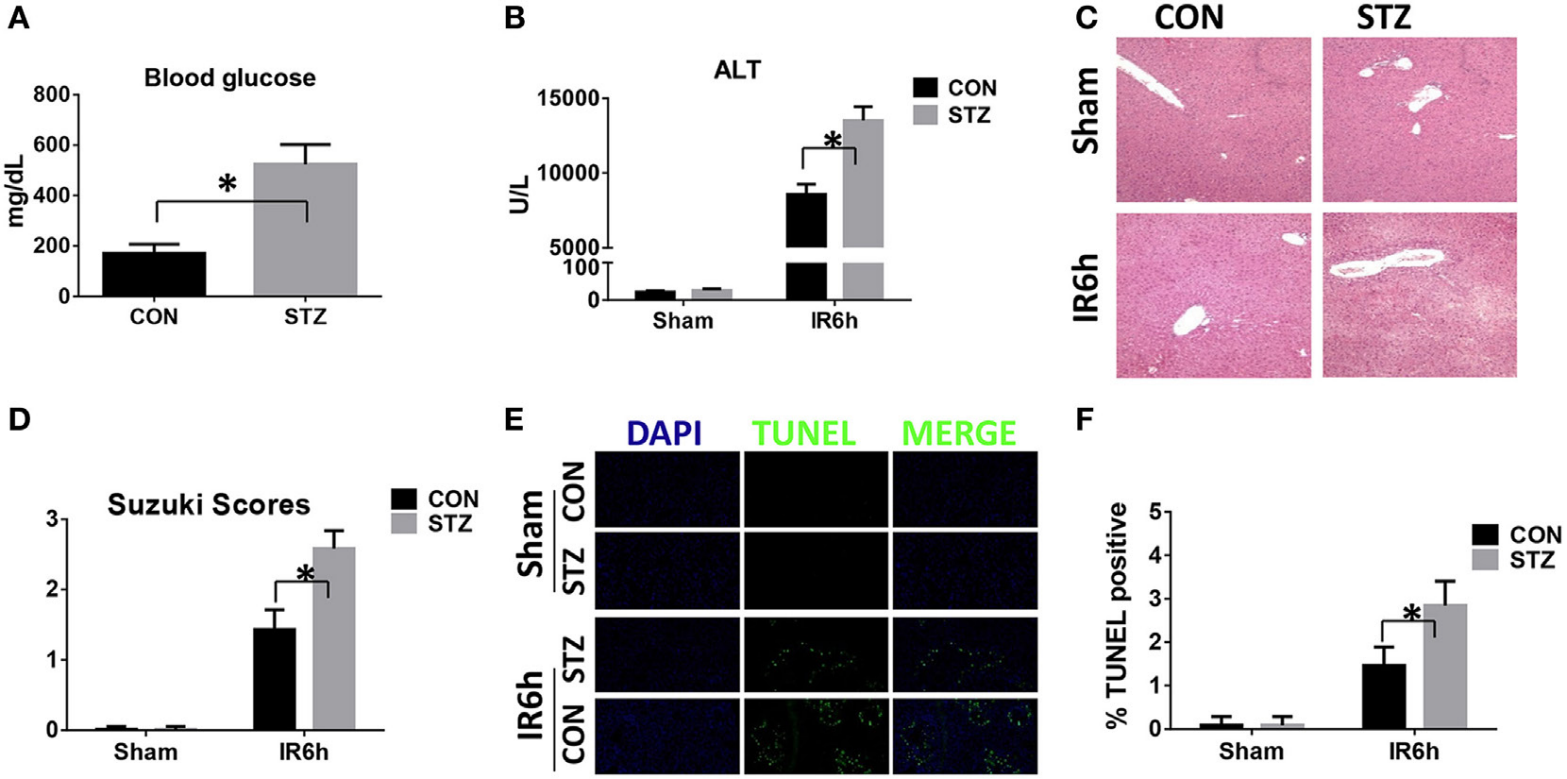
Hyperglycemia aggravates liver ischemia and reperfusion (IR) injury. Diabetic [streptozotocin (STZ)] and control (CON) mice were prepared as described in Section “Materials and Methods.” Liver partial warm IR or a sham procedure was performed. Blood glucose levels were measured in both groups [**(A)**, *n* = 6/group]. Liver injury was evaluated 6 h post-reperfusion in terms of serum ALT [**(B)**, *n* = 6/group], liver histopathology [**(C)**, results representative of six mice/group], and Suzuki scores [**(D)**, *n* = 6/group]. TUNEL staining of liver sections at 6 h after IR (original magnification ×20). DAPI was used for nuclear staining. Results representative of six mice/group **(E)**. Ratios of TUNEL-positive cells in different groups [**(F)**, *n* = 6/group] (*p < 0.05).

We first tested whether liver IR injury was aggravated by diabetes/hyperglycemia. Type I diabetes was induced by STZ and hyperglycemia was confirmed as shown in Figure 1A. Indeed, compared with CON groups, mice in the STZ groups developed significantly more severe liver IR injury at 6 h post-reperfusion, as demonstrated by higher levels of serum ALT (Figure 1B), severely damaged liver architecture (Figure 1C) with higher Suzuki scores (Figure 1D), and extensive hepatocellular apoptosis (Figures 1E,F). Thus, diabetes/hyperglycemia increased liver IR injury.

### Hyperglycemia-Enhanced KC-Related Inflammation after IR

**Figure 2:**
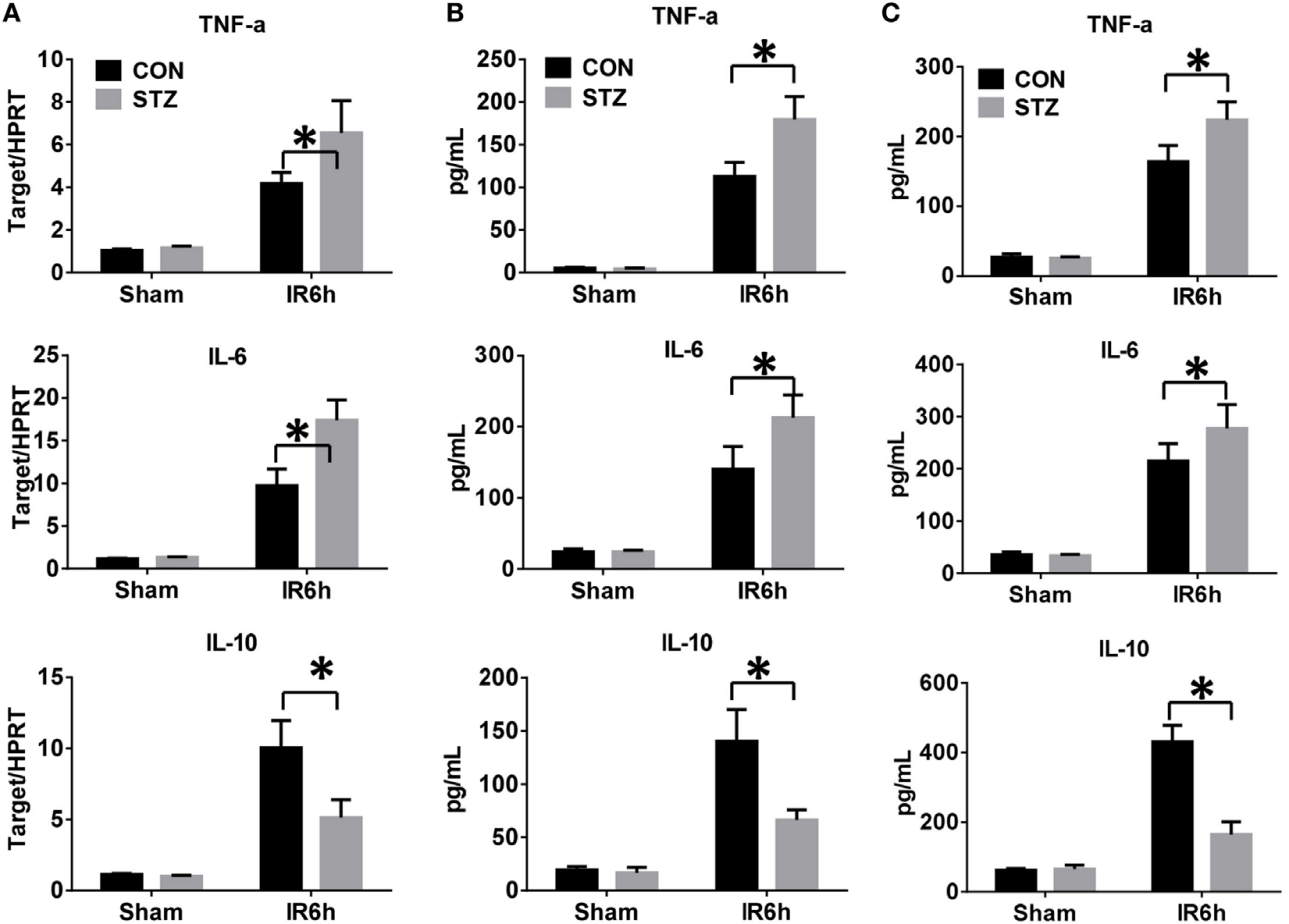
Hyperglycemia enhances Kupffer cell (KC)-related inflammation after ischemia and reperfusion (IR). Liver partial warm IR or a sham procedure was performed in diabetic [streptozotocin (STZ)] and control (CON) mice. Six hours post-reperfusion, inflammatory gene expression in liver tissues was evaluated by quantitative RT-PCR [**(A)**, *n* = 6/group]. Serum levels of inflammatory cytokines were measured by ELISA [**(B)**, *n* = 6/group]. KCs from CON or STZ mice following IR or the sham procedure were cultured *in vitro* for 6 h. TNF-α, IL-6, and IL-10 protein levels in the culture supernatant were measured by ELISA [**(C)**, *n* = 6/group] (*p < 0.05).

Because the inflammatory response plays a critical role in mediating liver IR injury, we next detected the inflammatory cytokines gene induction in IR-stressed livers by qRT-PCR. Hyperglycemic livers showed significantly higher levels of pro-inflammatory TNF-α and IL-6, but lower levels of anti-inflammatory IL-10 gene induction, as compared to the control livers (Figure 2A). These results were further supported by similar serum protein levels of TNF-α, IL-6, and IL-10, as measured by ELISA (Figure 2B). To further determine whether KC-mediated innate immune activation was affected by hyperglycemia, KCs isolated from CON or STZ mice post-IR or post-sham procedure were plated and cultured *in vitro*. After 6 h, levels of TNF-α, IL-6, and IL-10 protein in KC cultural supernatant were measured by ELISA. As shown in Figure 2C, the levels of pro-inflammatory TNF-α and IL-6 secreted by KCs were higher in the STZ group. Notably, the most significant difference between the CON and STZ groups was that KCs isolated from hyperglycemic mice secreted significantly lower levels of anti-inflammatory IL-10.

### Hyperglycemia-Enhanced ER Stress in Livers Post-IR

**Figure 3:**
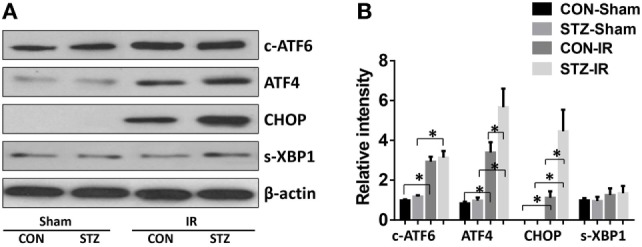
Hyperglycemia enhances endoplasmic reticulum stress in livers post-IR. Diabetic [streptozotocin (STZ)] and control (CON) mice were prepared and liver partial warm ischemia and reperfusion (IR) or sham procedure was performed. Six hours post-reperfusion, liver tissues were collected, and c-ATF6, ATF4, C/EBP homologous protein (CHOP), s-XBP1, and β-actin protein levels were analyzed by Western blotting. Representative of three experiments **(A)**. Relative density ratios of target proteins in different groups to the control group (CON-Sham) were calculated [**(B)**, *n* = 3/group] (*p < 0.05).

To evaluate whether ER stress was involved in the elevated IR injury in hyperglycemic livers, liver tissues from the CON and STZ groups were collected post-IR and analyzed by Western blotting (Figures 3A,B). Indeed, IR triggered ER stress in livers of both the CON and STZ groups, as demonstrated by significantly increased levels of c-ATF6, ATF4, and CHOP. However, s-XBP1 levels increased slightly but without significance. Interestingly, compared to the CON group, only ATF4 and CHOP levels were further increased in the STZ group, while no significant differences were found regarding c-ATF6 and s-XBP1 levels in the ischemic livers. These results indicated that the ATF4/CHOP signaling pathway might be involved in the detrimental effect of hyperglycemia in liver IR injury.

### CHOP-Mediated Hyperglycemic KC Pro-inflammatory Activation *In Vitro*

**Figure 4:**
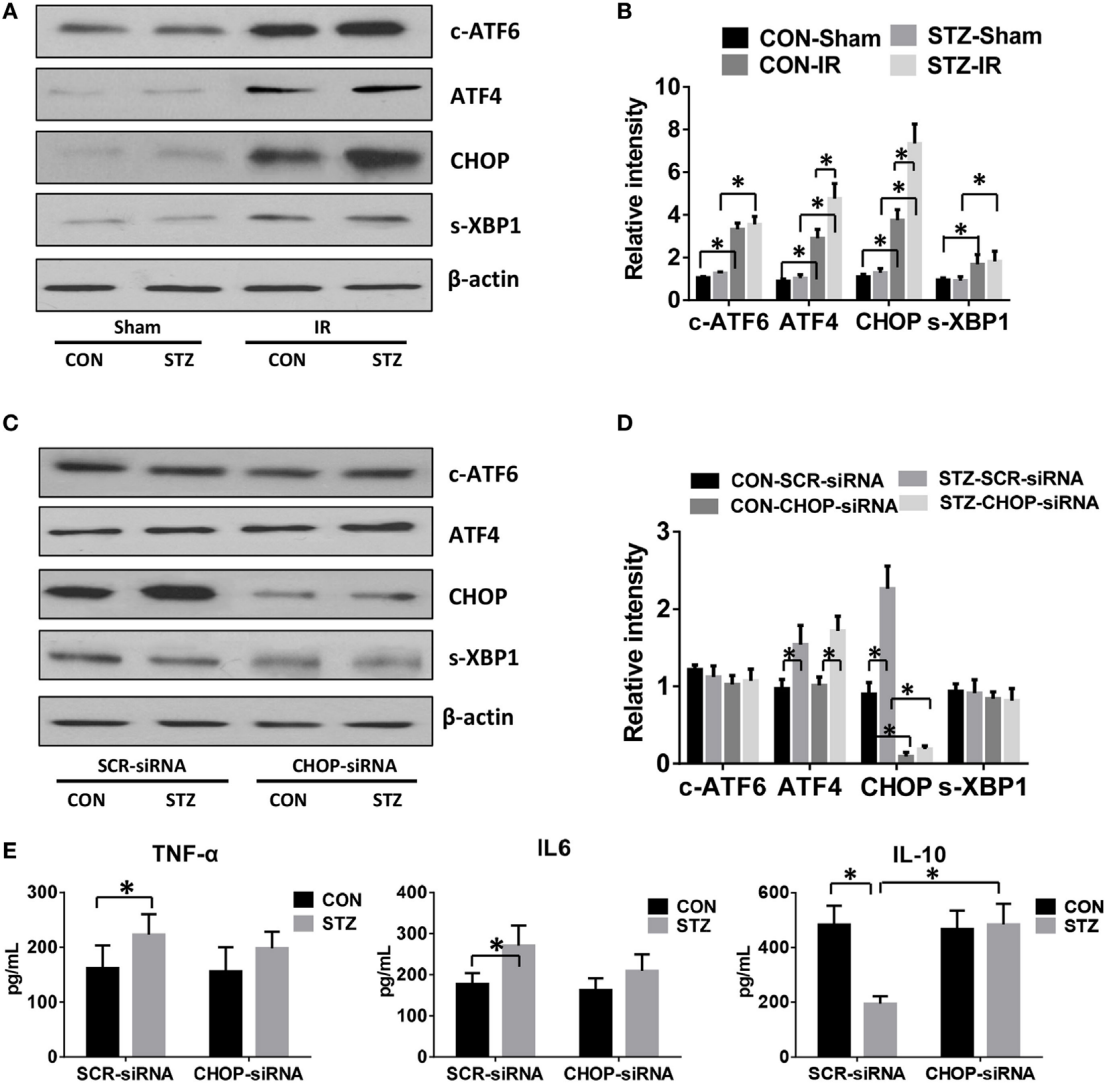
C/EBP homologous protein (CHOP) mediates hyperglycemic Kupffer cell (KC) pro-inflammatory activation *in vitro*. Diabetic [streptozotocin (STZ)] and control (CON) mice were prepared and liver partial warm ischemia and reperfusion (IR) or a sham procedure was performed. After 6 h of reperfusion, KCs were isolated and the intracellular levels of c-ATF6, ATF4, CHOP, s-XBP1, and β-actin protein were analyzed by Western blotting. Representative of three experiments **(A)**. Relative density ratios of target proteins in different groups to the control group (CON-Sham) were calculated [**(B)**, *n* = 3/group]. Both CON and STZ mice were pretreated with CHOP siRNA (CHOP-siRNA) or its scramble control siRNA (SCR-siRNA) *in vivo* prior to IR using mannose-conjugated polymers as described in Section “Materials and Methods.” Liver IR was performed. Six hours post-reperfusion, KCs were isolated and the intracellular levels of c-ATF6, ATF4, CHOP, s-XBP1, and β-actin protein were analyzed by Western blotting. Representative of three experiments **(C)**. Relative density ratios of target proteins in different groups to the control group (CON–SCR-siRNA) were calculated [**(D)**, *n* = 3/group]. Isolated KCs from IR-stressed livers of different groups were cultured for 6 h, and TNF-α, IL-6, and IL-10 protein levels in the culture supernatant were measured by ELISA [**(E)**, *n* = 6/group] (*p < 0.05).

Because hyperglycemia enhanced KC pro-inflammatory activation and intrahepatic ER stress, we proceeded to determine whether hyperglycemia regulates KC innate immune activation *via* ER stress signaling pathways. KCs were isolated from post-IR livers in both the CON and STZ groups, and intracellular ER stress markers were analyzed by Western blot. Our findings were consistent with those shown in Figure 3; while ATF6, ATF4/CHOP, and XBP1 in KCs from both the CON and STZ groups were activated by IR, ATF4/CHOP but not c-ATF6 and s-XBP1 levels were further increased in the STZ group compared to the CON group (Figures 4A,B, IR, CON vs. STZ).

To further determine the role of ATF4/CHOP signaling in regulating KC activation by hyperglycemia, we utilized mannose-conjugated polymers to deliver CHOP siRNA (CHOP-siRNA) or its scramble control siRNA (SCR-siRNA) *in vivo* before IR in both CON and STZ mice. Indeed, CHOP siRNA effectively inhibited CHOP activation in KCs in both the CON and STZ groups (Figures 4C,D). Furthermore, as shown in Figure 4E, CHOP knockdown slightly decreased pro-inflammatory TNF-α and IL-6 secretion by KCs in both CON and STZ mice. However, the most important effect of CHOP knockdown was on anti-inflammatory IL-10 secretion. CHOP knockdown dramatically increased the level of IL-10 level in KCs from the STZ group (STZ group, SCR-siRNA vs. CHOP-siRNA). By contrast, no significant difference was found in the CON group (CON group, SCR-siRNA vs. CHOP-siRNA).

### CHOP-Regulated Hyperglycemic KC M1/M2 Polarization

**Figure 5:**
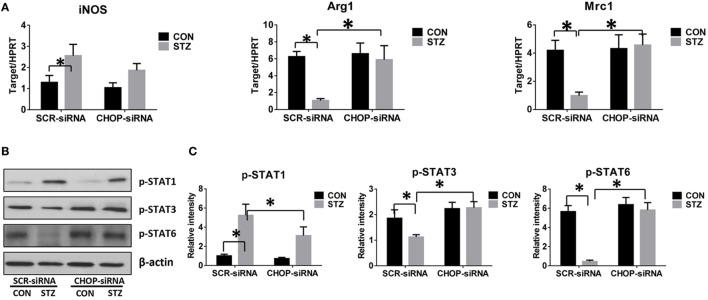
C/EBP homologous protein (CHOP)-regulated hyperglycemic Kupffer cell (KC) M1/M2 polarization. Both control (CON) and diabetic (STZ) mice were pretreated with CHOP siRNA (CHOP-siRNA) or its scramble control siRNA (SCR-siRNA) *in vivo* prior to ischemia and reperfusion. Six hours post-reperfusion, KCs were isolated, and *iNOS, Arg1*, and *Mrc1* gene induction was measured by quantitative RT-PCR [**(A)**, *n* = 6/group]. The intracellular levels of p-STAT1, p-STAT3, p-STAT6, and β-actin protein were analyzed by Western blotting. Representative of six experiments **(B)**. Relative density ratios of target proteins in different groups to the control group (CON-SCR-siRNA) were calculated [**(C)**, *n* = 6/group] (*p < 0.05).

Macrophages can be differentiated into classical pro-inflammatory M1 or alternative immune regulatory M2 phenotypes with distinct functions. We therefore evaluated the role of CHOP signaling in regulating macrophage M1/M2 polarization by hyperglycemia. Indeed, KCs isolated from hyperglycemic mice exhibited higher levels of *iNOS* but much lower levels of *Arg1* and *Mrc1* gene induction (Figure 5A). Furthermore, as shown by Western blot in Figures 5B,C, hyperglycemic KCs were marked by increased activation of STAT1 but decreased STAT3 and STAT6 activation post-IR.

Interestingly, CHOP knockdown significantly enhanced *Arg1* and *Mrc1* gene expression in KCs isolated from the STZ group but had no remarkable effects on the CON group. While *iNOS* gene induction was slightly decreased by CHOP knockdown in KCs from both the CON and STZ groups (Figure 5A), no significant changes in STAT1, STAT3, and STAT6 phosphorylation were observed following CHOP knockdown in the KCs isolated from CON group mice. By contrast, CHOP knockdown in KCs from the STZ group resulted in decreased activation of STAT1, and more importantly, restored the activation of both STAT3 and STAT6 (Figures 5B,C). Thus, hyperglycemia inhibited KC M2 differentiation post-IR. Over-activation of CHOP signaling was essential for the inhibition of IL-10-secreting M2 differentiation, thereby resulting in excessive inflammation during liver IR.

### CHOP-Mediated Hyperglycemic KC Hyper-Inflammatory Immune Activation and Accelerated Liver IR Injury *In Vivo*

**Figure 6:**
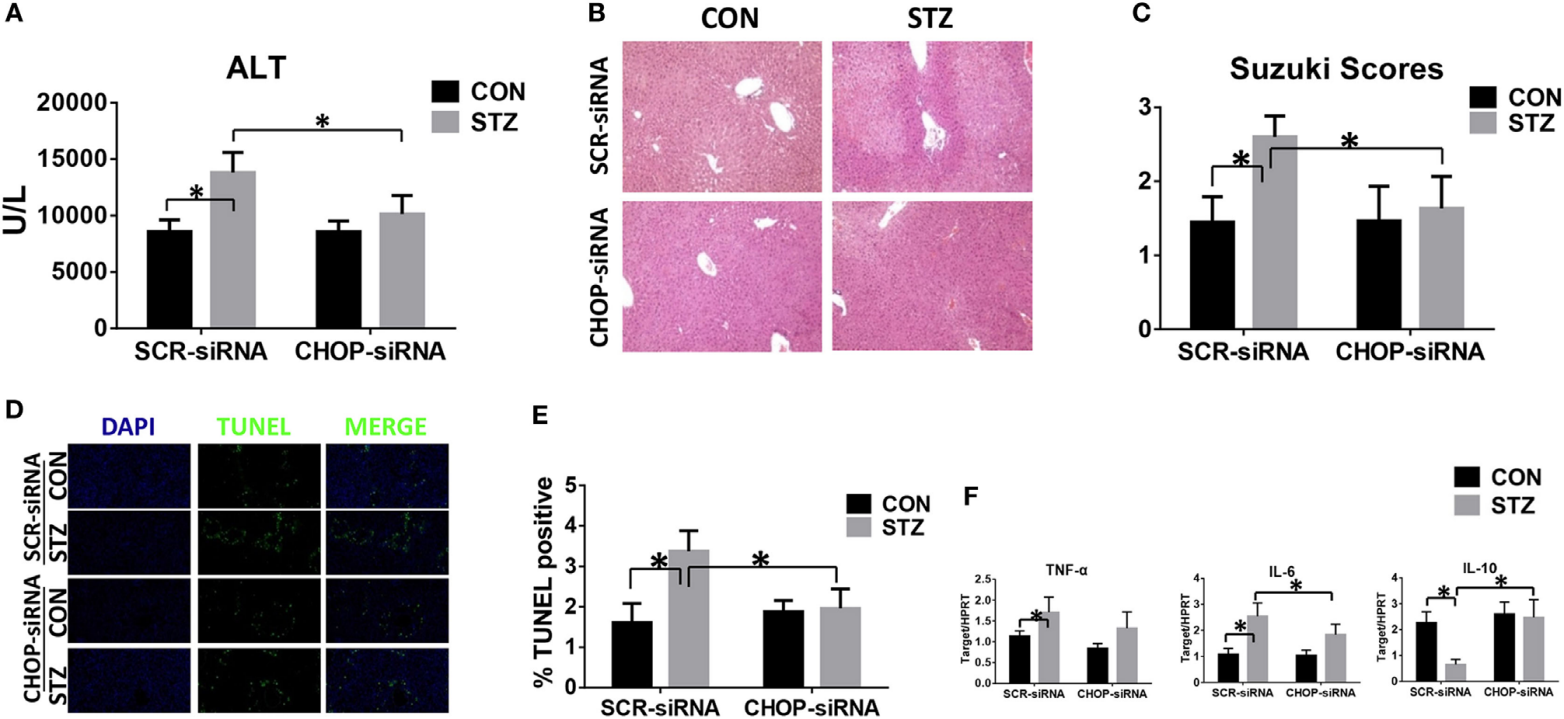
C/EBP homologous protein (CHOP) mediates hyperglycemic Kupffer cell hyper-inflammatory immune activation and accelerates liver ischemia and reperfusion (IR) injury *in vivo*. Both CON and streptozotocin (STZ) mice were pretreated with CHOP siRNA (CHOP-siRNA) or its scramble control siRNA (SCR-siRNA) *in vivo* prior to IR using mannose-conjugated polymers as described in Section “Materials and Methods.” Liver injury was evaluated 6 h post-reperfusion in terms of serum ALT [**(A)**, *n* = 6/group], liver histopathology **(B)**, results representative of six mice/group, and Suzuki scores [**(C)**, *n* = 6/group]. TUNEL staining of liver sections at 6 h after IR (original magnification ×20). DAPI was used for nuclear staining. Results representative of six mice/group **(D)**. Ratios of TUNEL-positive cells in different groups [**(E)**, *n* = 6/group]. Inflammatory gene expression in liver tissues was evaluated by quantitative RT-PCR [**(F)**, *n* = 6/group] (*p < 0.05).

Finally, we studied the functional significance of CHOP signaling in regulating KC innate immune activation during hyperglycemic liver IR injury. CHOP signaling in KCs was knocked down *in vivo* by CHOP-siRNA treatment prior to ischemia. Interestingly, *in vivo* CHOP knockdown had no significant effects on liver IR injury in the CON group. By contrast, CHOP knockdown protected hyperglycemic livers against IR injury, as evidenced by lower levels of serum ALT (Figure 6A), better preserved liver architecture (Figure 6B) with lower Suzuki scores (Figure 6C) and less hepatocellular apoptosis (Figures 6D,E). Furthermore, while CHOP gene knockdown *in vivo* had no effect on intrahepatic TNF-α, IL-6, and IL-10 gene induction in the CON group, it decreased pro-inflammatory TNF-α and IL-6 gene induction and, most importantly, restored anti-inflammatory IL-10 gene induction in livers post-IR in the STZ group (Figure 6F).

## Discussion

Diabetes mellitus is very common in patients undergoing hepatic surgery. However, the perioperative blood glucose level is usually well controlled to reduce related postoperative complications. Thus, a hyperglycemic mouse model is needed to better study the role and regulatory mechanism of hyperglycemia in regulating liver IR injury. In the present study, a hyperglycemic mouse model is established by STZ treatment. Our study identifies a novel mechanism by which hyperglycemia aggravates liver IR injury. Hyperglycemia induces the over-activation of CHOP-mediated ER stress in KCs, which in turn inhibits the anti-inflammatory M2 polarization of KCs, thereby leading to excessive intrahepatic inflammation and ultimately greater hepatocellular injury.

Two distinctive stages of liver IR injury have been defined. In comparison with the direct hepatocellular damage caused by oxygen and nutrition depletion upon hepatic portal occlusion during ischemia, subsequent intrahepatic inflammation resulted in more extensive liver injury. The pathogen-associated molecular patterns or damage-associated molecular patterns following liver ischemic stress activate innate immune cells through pattern recognition receptors, thereby playing a critical role in the pathogenesis of liver IR injury (2). Consistent with other studies, we found that hyperglycemia aggravates liver IR injury by inducing the over-activation of intrahepatic inflammation (9, 10).

Kupffer cells, the liver-resident macrophages, are abundant in the liver and make up more than 50% of all resident macrophages and 15% of all hepatic cells (23). The pro-inflammatory roles of KCs during IR have been reported in many studies. However, while both liver-resident KCs and infiltrating macrophages are involved in the immune response against liver IR, the majority of previous studies do not distinguish between these types of macrophages. Studies using clodronate liposomes to achieve specific depletion of KCs have revealed their anti-inflammatory effects (24, 25). Furthermore, recent studies have highlighted the marked heterogeneity of tissue macrophages arising from hematopoietic versus self-renewing embryo-derived populations (26, 27). Under hyperglycemic conditions, a significant increase in the infiltration of the liver, kidney, and intestines by macrophages has been reported, thereby leading to increased host vulnerability to new inflammatory challenges (13). In the present study, we found that in control mice, post-IR KCs release not only pro-inflammatory TNF-α and IL-6 but also anti-inflammatory IL-10. The activation of protective IL-10 during the macrophage pro-inflammatory immune response may play an important role in the autoregulation of excessive inflammation under stress. By contrast, in hyperglycemic mice, the secretion of anti-inflammatory IL-10 by KCs was significantly inhibited, thereby leading to uncontrolled inflammation and aggravated liver IR injury. These findings also indicate a protective role for KCs in controlling the hyper-inflammatory immune response *via* IL-10 expression during liver IR injury.

Endoplasmic reticulum stress is a cellular adaptive response that resolves organelle dysfunction and ensures survival under stressful conditions. UPR is activated upon ER stress to inhibit new protein synthesis and activate the transcription of genes encoding proteins involved in protein folding and degradation in the ER (14). Accumulating data have demonstrated the crucial functions of ER stress in immunity, inflammation, and various prevalent diseases, including inflammatory bowel disease, metabolic disease, cancer, and liver disease (28, 29). The induction of ER stress in macrophages strongly potentiates LPS-induced pro-inflammatory chemokine and cytokine expression (30, 31). Toll-like receptor (TLR) signaling induces ER stress, which in turn amplifies macrophage activation. Activation of the UPR together with TLR4 stimulation results in the synergistic enhancement of TLR4 inflammatory responses. TLR4 and TLR2 specifically activate the ER stress sensor kinase IRE1α and its downstream target, the transcription factor XBP1. In mice, macrophage-specific XBP1 deficiency impairs the production of IL-6, TNF, and interferon-β, thereby leading to a much greater bacterial burden (32). The PERK/ATF4 pathway has also been shown to induce inflammation through the direct binding of ATF4 to the IL-6 promoter. Both pharmacological activation and overexpression of ATF4 enhance IL-6 expression in macrophages (33). In our hyperglycemic IR model, we found that all three branches of the UPR signaling pathway were activated by IR, as indicated by increased levels of c-ATF6, ATF4, CHOP, and s-XBP1. Interestingly, ATF4 and CHOP, but not ATF6 and s-XBP1, were further enhanced by hyperglycemia compared to the CON group (Figures 3A and 4A). These results suggest that the ATF4/CHOP signaling pathway might be involved in regulating the hyper-inflammatory activation of KCs.

C/EBP homologous protein was first reported as a molecule involved in ER stress-induced apoptosis (34), and recent studies have indicated its critical roles in inflammatory responses (35). LPS treatment in mice activated the UPR and induced CHOP activation (36). By contrast, CHOP knockout suppressed LPS-induced caspase-11 expression and lung inflammation. Moreover, LPS-induced IL-1β secretion was attenuated in CHOP knockout mice (37). CHOP knockout also inhibited ER stress-induced IL-6 expression in macrophages, resulting in more rapid recovery from dextran sodium sulfate colitis (38). In our study, CHOP gene knockdown reduced TNF-α and IL-6 secretion in hyperglycemic KCs post-LPS stimulation. More importantly, inhibition of CHOP over-activation in hyperglycemia KCs post-IR restored anti-inflammatory IL-10 production. By contrast, a protective role for CHOP was reported in an LPS-induced inflammation and kidney injury model; CHOP deficiency resulted in increased NF-κB activation and severe inflammation (39). Several studies also found that TLR signaling inhibited ATF4/CHOP gene induction. TRIF-mediated signals from TLRs selectively attenuate translational activation of ATF4 and its downstream target gene CHOP. CHOP suppression by TLR might be required for promoting the survival of prolonged ER-stressed macrophages during immune activation (40, 41).

Macrophages are typically categorized into M1 or M2 phenotypes depending on the cytokines released, cell surface markers, and transcriptional profiles over the course of adaptation to the local microenvironment during the progression of liver injury (42). While M1 macrophages predominantly exert a pro-inflammatory role during liver injury, M2 macrophages play an anti-inflammatory or pro-fibrotic role during liver repair and fibrosis. Previous studies have demonstrated the influence of hyperglycemia in macrophage M1/M2 polarization. In primary human monocyte-derived macrophages, enhanced M1-like inflammatory polarization was observed upon exposure to high levels of glucose, both under *in vitro* culture conditions and in patients with hyperglycemia (43). In another study of human macrophages, hyperglycemia enhanced the expression of the M1 cytokines TNF-α and IL-1β as well as M2 cytokine IL-1Ra expression. Meanwhile, the expression of CCL18 was suppressed in M2 macrophages by hyperglycemia (44). In the ischemic stroke model, hyperglycemia switched monocytes/macrophages from a pro-inflammatory to a non-inflammatory polarization and increased the infarct volume (45). However, the precise mechanism of hyperglycemia in regulating macrophage M1/M2 polarization remains to be defined. The signal transducers and activators of transcription (STATs), peroxisome proliferator-activated receptor family members, and hypoxia-inducible factor 2α have been shown to regulate macrophage polarization (46). A recent study showed that KCs from STZ-induced hyperglycemic mice expressed higher levels of receptor of advanced glycation end product (RAGE), thereby leading to hyper-inflammatory immune responses and increased liver IR injury (9). The RAGE signaling pathway was also found to be involved in regulating monocytes/macrophages in stroke brains (45). Interestingly, increasing studies suggest that ER stress and CHOP signaling could be upregulated by RAGE signaling in various cell types (47–50). These findings inspired us to further characterize the role of CHOP-mediated ER stress in regulating KC activation under hyperglycemic conditions.

There is some controversy surrounding the effect of CHOP signaling in regulating macrophage M1/M2 polarization (21, 51–53). One recent study found that a high-fat diet enhanced ER stress and upregulated CHOP expression in adipocytes. CHOP deficiency induced adipose tissue macrophage polarization to alternatively activate the M2 macrophage phenotype (21). By contrast, CHOP was found to exacerbate allergic airway inflammation by enhancing M2 programming in macrophages in an ovalbumin-induced allergic airway inflammation model (52). Similarly, CHOP deficiency protected mice against bleomycin-induced pulmonary fibrosis by attenuating M2 macrophage production (53). Thus, the functions of CHOP signaling in regulating inflammation may vary depending on the cell type and stress setting. In the current study, we evaluated CHOP activation in KCs post-IR and found that CHOP protein levels were increased by IR and were further enhanced in STZ-induced hyperglycemic mice. Over-activation of CHOP in hyperglycemic KCs was associated with inhibition of the M2 KC phenotype, as evidenced by decreased *Arg1* and *Mrc1* gene induction, inhibited STAT3 and STAT6 activation, and much lower levels of IL-10 production. CHOP-siRNA restored IL-10 expression and the M2 phenotype in hyperglycemic KCs.

In conclusion, our study demonstrates that hyperglycemia restrains the KC anti-inflammatory response during liver IR injury. Hyperglycemia-induced CHOP over-activation inhibited M2 polarization and suppressed IL-10 production, which is responsible for the hyper-inflammatory immune response and exacerbated liver IR injury observed in diabetic/hyperglycemic hosts. This mechanism may constitute a potential therapeutic target for liver IR injury.

## Ethics Statement

All animals received humane care and all animal procedures met the relevant legal and ethical requirements according to a protocol (number NMU08-092) approved by the Institutional Animal Care and Use Committee of Nanjing Medical University.

## Author Contributions

CL, ZD, and ZR designed the research; ZR, JS, XP, ZC, and HS performed the experiments; PZ and MG analyzed the data; ZR wrote the manuscript.

## Conflict of Interest Statement

The authors declare that the research was conducted in the absence of any commercial or financial relationships that could be construed as a potential conflict of interest.
